# Comparison of pharmaceutical properties and biological activities of prednisolone, deflazacort, and vamorolone in DMD disease models

**DOI:** 10.1093/hmg/ddad173

**Published:** 2023-10-11

**Authors:** Grace Liu, Philip Lipari, Anna Mollin, Stephen Jung, Irina Teplova, Wencheng Li, Lanqing Ying, Vijay More, William Lennox, Shirley Yeh, Eric McGann, Young-Choon Moon, Cari Rice, Eduardo Huarte, Barbara Gruszka, Balmiki Ray, Elizabeth Goodwin, Patricia Buckendahl, Edward Yurkow, Bruce Braughton, Jana Narasimhan, Ellen Welch, Gregory Voronin, Marla Weetall

**Affiliations:** PTC Therapeutics, Inc., 100 Corporate Court, South Plainfield, NJ 07080, United States; PTC Therapeutics, Inc., 100 Corporate Court, South Plainfield, NJ 07080, United States; PTC Therapeutics, Inc., 100 Corporate Court, South Plainfield, NJ 07080, United States; PTC Therapeutics, Inc., 100 Corporate Court, South Plainfield, NJ 07080, United States; PTC Therapeutics, Inc., 100 Corporate Court, South Plainfield, NJ 07080, United States; PTC Therapeutics, Inc., 100 Corporate Court, South Plainfield, NJ 07080, United States; PTC Therapeutics, Inc., 100 Corporate Court, South Plainfield, NJ 07080, United States; PTC Therapeutics, Inc., 100 Corporate Court, South Plainfield, NJ 07080, United States; PTC Therapeutics, Inc., 100 Corporate Court, South Plainfield, NJ 07080, United States; PTC Therapeutics, Inc., 100 Corporate Court, South Plainfield, NJ 07080, United States; PTC Therapeutics, Inc., 100 Corporate Court, South Plainfield, NJ 07080, United States; PTC Therapeutics, Inc., 100 Corporate Court, South Plainfield, NJ 07080, United States; PTC Therapeutics, Inc., 100 Corporate Court, South Plainfield, NJ 07080, United States; PTC Therapeutics, Inc., 100 Corporate Court, South Plainfield, NJ 07080, United States; PTC Therapeutics, Inc., 100 Corporate Court, South Plainfield, NJ 07080, United States; PTC Therapeutics, Inc., 100 Corporate Court, South Plainfield, NJ 07080, United States; PTC Therapeutics, Inc., 100 Corporate Court, South Plainfield, NJ 07080, United States; Rutgers University, Molecular Imaging Center, 41 Gordon Road, Piscataway, NJ 08854, United States; Rutgers University, Molecular Imaging Center, 41 Gordon Road, Piscataway, NJ 08854, United States; PTC Therapeutics, Inc., 100 Corporate Court, South Plainfield, NJ 07080, United States; PTC Therapeutics, Inc., 100 Corporate Court, South Plainfield, NJ 07080, United States; PTC Therapeutics, Inc., 100 Corporate Court, South Plainfield, NJ 07080, United States; PTC Therapeutics, Inc., 100 Corporate Court, South Plainfield, NJ 07080, United States; PTC Therapeutics, Inc., 100 Corporate Court, South Plainfield, NJ 07080, United States

**Keywords:** Duchenne muscular dystrophy, deflazacort, prednisolone, corticosteroids, PK-PD

## Abstract

Duchenne muscular dystrophy (DMD) is a progressive disabling X-linked recessive disorder that causes gradual and irreversible loss of muscle, resulting in early death. The corticosteroids prednisone/prednisolone and deflazacort are used to treat DMD as the standard of care; however, only deflazacort is FDA approved for DMD. The novel atypical corticosteroid vamorolone is being investigated for treatment of DMD. We compared the pharmaceutical properties as well as the efficacy and safety of the three corticosteroids across multiple doses in the B10-mdx DMD mouse model. Pharmacokinetic studies in the mouse and evaluation of p-glycoprotein (P-gP) efflux in a cellular system demonstrated that vamorolone is not a strong P-gp substrate resulting in measurable central nervous system (CNS) exposure in the mouse. In contrast, deflazacort and prednisolone are strong P-gp substrates. All three corticosteroids showed efficacy, but also side effects at efficacious doses. After dosing mdx mice for two weeks, all three corticosteroids induced changes in gene expression in the liver and the muscle, but prednisolone and vamorolone induced more changes in the brain than did deflazacort. Both prednisolone and vamorolone induced depression-like behavior. All three corticosteroids reduced endogenous corticosterone levels, increased glucose levels, and reduced osteocalcin levels. Using micro-computed tomography, femur bone density was decreased, reaching significance with prednisolone. The results of these studies indicate that efficacious doses of vamorolone, are associated with similar side effects as seen with other corticosteroids. Further, because vamorolone is not a strong P-gp substrate, vamorolone distributes into the CNS increasing the potential CNS side-effects.

## Introduction

Duchenne muscular dystrophy (DMD) is a progressive disabling neuromuscular X-linked recessive disorder that affects about 1:3500–1:5000 boys worldwide [1, 2]. DMD arises from a mutation in the dystrophin gene that results in a deficiency of functional dystrophin protein. Dystrophin is critical to the structural and membrane stability of muscle fibers in skeletal and cardiac muscle [3, 4]. The loss of functional dystrophin results in inflammation and progressive replacement of muscle fibers with fat and connective tissue, causing gradual and irreversible loss of muscle function [3–5].

Patients with DMD typically lose the ability to walk by their early teens, require ventilation support in their late teens, and, eventually, die due to heart and/or respiratory failure with a median survival time of 30 years [6, 7]. Moreover, approximately one third of these patients present with behavioral and cognitive impairments including intellectual disability, attention deficit hyperactivity disorder, and autism spectrum disorders [8, 9].

The corticosteroids prednisone/prednisolone and deflazacort are the current standard of care for treating DMD [10] and prolong reduction in muscle strength and the time before loss of ambulation and mortality in DMD patients [11–13]. While both prednisone/prednisolone and deflazacort are used to treat patients with DMD, only deflazacort is FDA approved for this purpose. Evidence from randomized clinical trials indicated that corticosteroids improve muscle function in DMD, delaying the time to loss of ambulation relative to no treatment with corticosteroids [14]. In subsequent studies, corticosteroid use in non-ambulatory patients with DMD was associated with delayed progression of pulmonary, cardiac, and upper limb loss of function when compared with those who did not receive corticosteroids [15]. Deflazacort is administered as a prodrug that forms the active metabolite 21-des deflazacort in plasma. Prednisone is also a prodrug, forming the active metabolite prednisolone in the plasma.

High doses or chronic corticosteroid use is associated with significant adverse effects that can limit their long-term use including excessive weight gain, adrenal insufficiency, behavioral changes, Cushing’s syndrome, stunted growth, and osteoporosis [16–18]. Psychiatric disorders induced by chronic steroid use range in severity and can include depression, insomnia, mania, panic attacks, and even psychosis [19, 20]. Exacerbation of DMD-associated behavioral problems with corticosteroids complicate corticosteroid use.

**Figure 1:**
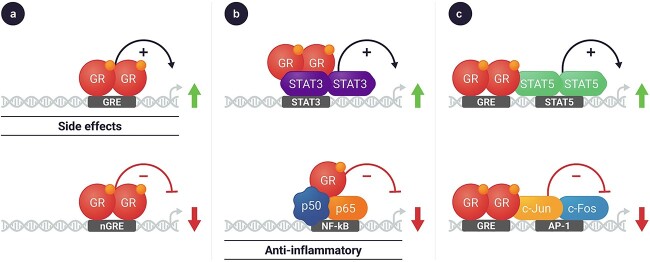
Corticosteroid-activated glucocorticoid receptors regulate gene expression via multiple mechanisms. Glucocorticoid ligands bind to the glucocorticoid receptor in the cytosol. The complex traverses to the nucleus where it can regulate gene expression by a variety of mechanisms including activation and suppression, such as (a) directly binding to GRE as a dimer promoting transcription of genes that may be associated with side effects (upper panel), and negatively regulates genes such as collagen, POMC gene expression (lower panel); (b) tethering to other proteins that are binding to the DNA to regulate gene expression, such as STAT3, to turn on DNA methylation and histone modifications (top), or by interacting and inhibiting NF-kB activity that is associated with anti-inflammatory effects (bottom); or (c) binding directly to DNA and interacting with neighboring transcription factors, such as STAT5 (top) and c-Jun and c-Fos (bottom).

Corticosteroids act by binding to the glucocorticoid receptor (GR). The GR-ligand complex subsequently migrates from the cytosol to the nucleus. There, the complex acts by at least two different mechanisms to influence the transcription of multiple genes [21] (Fig. 1). Many of the anti-inflammatory effects of corticosteroids results from the association of the GR-ligand complex with other proteins, including the trans-suppression of transcription of the proinflammatory nuclear factor kappa B (NF-κB) [21]. A second mechanism by which corticosteroids act is via direct binding of the GR-ligand complex to the glucocorticoid-response element (GRE) to directly affect the transcription of multiple genes [21]. This latter, direct GRE-mediated transcriptional regulation is generally associated with the adverse effects that are attributed to corticosteroid therapy [21]. Overall, corticosteroids result in extensive changes in gene expression, resulting in alterations in multiple physiological processes including mood and cognitive function, immune response, and metabolism [18, 21].

The novel corticosteroid, vamorolone, is being investigated for the treatment of DMD and is described as an anti-inflammatory steroid with fewer side-effects. Vamorolone has been reported to have favorable anti-inflammatory activity by inhibiting NF-κB while being “dissociated” from side effects by not activating GRE-mediated transcripts. Vamorolone lacks a 11β-hydroxyl/carbonyl moiety on the steroidal C-ring resulting in the loss of one of the five known sites of corticosteroid ligand binding to the glucocorticoid receptor [22]. Because of this altered interaction with the glucocorticoid receptor, the vamorolone-glucocorticoid receptor complex shows reduced binding to GREs in cellular assays. Vamorolone therapy was reported to be efficacious with reduced side effects in the mdx mouse model of DMD [23, 24] and in DMD clinical trials [25–31]. The mdx mouse studies, which assessed doses between 5 and 45 mg/kg, found vamorolone promoted repair of skeletal muscle cells following laser injury through anti-inflammatory signaling and membrane stabilization pathways and had little effect on hormonal regulation, growth, or immunosuppression [24]. The drug was most active at doses ≥20 mg/kg [24]. Vamorolone was found to be a mineralocorticoid receptor antagonist, while prednisolone was shown to be a mineralocorticoid receptor agonist [23]. In contrast, deflazacort had no effect on mineralocorticoid receptor signaling [23].

In a Phase 2A clinical trial, boys, aged 4.5 to 7.5 years with DMD were treated for 2 weeks at 2 mg/kg or 6 mg/kg vamorolone followed by a 2-week washout period [27]. Following the washout period, subjects could join a 24-week extension dose finding study followed by a long-term 24-month extension study [28]. After 30 months of treatment of the combined dose finding and long-term extension studies, subjects treated with the higher dose vamorolone showed no statistically significant difference between the vamorolone treated patients and matched natural history control group receiving corticosteroid therapy in the North Star Ambulatory Assessment (NSAA) total score, timed-function tests, or body mass index [28]. Vamorolone up to 6.0 mg/kg/day was well tolerated. Subjects in the natural history control group showed significant growth delay compared with participants receiving vamorolone [28]. This was confirmed in Ph2B/3 trial comparing vamorolone and prednisolone. In that study, vamorolone showed equal or better efficacy and less stunting of growth than did prednisolone [30].

**Figure 2:**
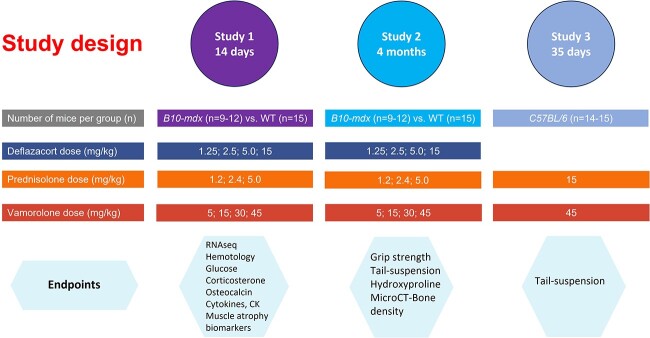
Study design. Study 1 was designed to evaluate the molecular side-effects of the different corticosteroids in the mdx mouse. Study 2 and Study 3 were aimed at evaluating CNS side effects and efficacy of the corticosteroids in the mdx mouse and wild-type mouse, respectively. The number of mice per group is included in parentheses. Abbreviations: CK; Creatine kinase; microCT, micro computed tomography.

Although deflazacort, prednisolone, and vamorolone have all shown efficacy in both preclinical mouse models of DMD and in clinical trials, no studies have systematically compared all three corticosteroids side by side for efficacy and safety. The objective of the series of studies reported here was to compare the efficacy and safety of the three corticosteroids across multiple doses (Fig. 2). We evaluated a range of biochemical and functional measures in three studies, two studies in B10-mdx mice comparing all 3 corticosteroids and one study in wildtype mice comparing vamorolone and prednisolone. Our results show that while all three corticosteroids reduce pathology in the mdx DMD mouse, at the doses associated with efficacy in the mdx mouse these drugs also exhibit biochemical changes associated with side-effects.

## Results

### Pharmacokinetics and biodistribution of prednisolone, deflazacort, and vamorolone in mice

The effect of corticosteroids on behavior may in part reflect drug exposure and subsequent regulation of gene transcription in the brain. To assess whether the three corticosteroids can cross the blood-brain barrier, we dosed by oral gavage prednisolone (the active metabolite of prednisone; administered in 0.1% hydroxypropyl methyl cellulose (HPMC) with 0.5% Tween 80 (pH 4.0), deflazacort (which forms the active metabolite 21-des-deflazacort in vivo; administered in 0.1% HPMC with 0.5% Tween 80 (pH 4.0)), and vamorolone (administered in cherry syrup as previously published; [23, 24]). Prednisolone, 21-des-deflazacort, and vamorolone concentrations were then measured in the plasma and brain of mice. The doses chosen for each corticosteroid were shown in preclinical studies to be efficacious in slowing the muscle degeneration in the B10-mdx mice [23, 32]. The brain-to-plasma ratio was then calculated using the area under the curve (AUC) for brain exposure and the AUC for plasma exposure.

The brain-to-plasma ratio of vamorolone was greater than that for deflazacort or prednisolone: ~0.04 for deflazacort, ~0.08 for prednisolone, and ~0.30 for vamorolone (Table 1), indicating vamorolone penetrates the brain to a greater extent than deflazacort and prednisolone. An additional study was done in C57BL/10 mice and similar data were obtained (Supplementary Fig. S1, Supplementary Table S1). The doses utilized represent approximately 0.6-, 0.7, and 8-fold the AUC and 0.7, 0.6, 3-fold the C_max_ of the reported clinical dose for deflazacort, prednisolone, and vamorolone, respectively (Supplementary Table S4; note, limited PK data reported for prednisolone in DMD so the comparator human data was extrapolated from PK measured at lower doses in adults). At 5 mg/kg vamorolone, the brain/plasma ratio measured in mice was similar to that measured at 30 mg/kg vamorolone (Supplementary Fig. S1, Supplementary Table S1). The difference among the three corticosteroids with respect to their levels in the brain may reflect a difference in the ability of the P-glycoprotein (P-gp) to pump these drugs from the CNS back into the blood. P-gp is an ATP-dependent drug transport protein expressed on the blood luminal membrane of the brain capillary endothelial cells that make up the blood-brain barrier and functions as a biological barrier by expelling small molecules out of the CNS.

**Table 1:** Prednisolone, deflazacort, and vamorolone differ in blood/brain biodistribution.

	Prednisolone (5 mg/kg)	Deflazacort (4 mg/kg)	Vamorolone (30 mg/kg)
Plasma AUC (hr∙ng/ml)	3929	357	26 590
Plasma C_max_ (ng/ml)			2698
Brain AUC (h∙ng/g of tissue)	300	13.5	8070
Brain C_max_ (ng/g of tissue)			1825
Brain/plasma ratio based on AUC	~0.08	~0.04	~0.30
Brain/plasma ratio based on C_max_			~0.68

Abbreviations: AUC, area under the time vs drug concentration curve; Cmax, maximum drug concentration. Drug concentrations reported for deflazacort represent the levels of the active metabolite.

To measure transport by P-gp, Madin-Darby canine kidney (MDCK) cells that had been transfected with the human P-gp (*MDR1*) gene were plated in a Transwell plate and cultured until polarized. MDCK cells that are not transfected with human P-gp were used as a control. Compounds were added to either the apical or the basolateral side of the cells, and transport across the membrane was measured. Compounds that are transported by P-gp will show a higher basolateral concentration than apical concentration, as the P-gp is expressed only on the apical side of the cells.

This MDR1-MDCK permeability assay showed that at concentration of 1 and 10 μM, 21-des-deflazacort, deflazacort, prednisone, and prednisolone are P-gp substrates and are pumped back across the cell membrane (high efflux ratios, low Pap values (<3 × **10**^**−6**^ **cm/s**), indicating a low brain penetration classification; Table 2). Vamorolone had a lower efflux ratio, indicating it is a weaker P-gp substrate (low efflux ratio, higher Papp values (>3 × **10**^**−6**^ **cm/s**), and moderate brain penetration classification; see Materials and Methods definition of brain penetration class, and Table 2). This would suggest that the lower concentration of deflazacort, 21-des-deflazacort, prednisone, prednisolone in the mouse brain is due to active P-gp at the blood-brain barrier. Vamorolone is subject to less P-gp efflux, allowing for greater penetration of drug in the CNS.

**Table 2:** P-gp efflux ratio measured using MDCK-MDR1 cells demonstrates that vamorolone is a weaker P-gp substrate than prednisone/prednisolone and Deflazacort/21-des-Deflazacort.

P-gp Efflux Ratio Measured using MDCK-MDR1 Cells					
	Concentration (μM)	MDCK Efflux Ratio	MDCK-MDRI Efflux Ratio	Differences between ratios	
Prednisone	10	1.37	104	103	
Prednisone	1	1.20	167	166	
Prednisolone	10	4.46	143	139	
Prednisolone	1	2.01	69.7	68	
Deflazacort	10	1.01	58.4	57	
Deflazacort	1	0.949	60.2	59	
21-Des-deflazacort	10	1.40	66.3	65	
21-Des-deflazacort	1	1.24	67.4	66	
Vamorolone	10	0.746	6.42	6	
Vamorolone	1	0.755	6.84	6	
**Transport Across MDCK and MDR1-MDCK Cell Layers**					
	**Concentration (μM)**	**Apical to Basolateral MDCK (10** ^**−6**^ **cm/s)**	**Basolateral to Apical MDCK (10** ^**−6**^ **cm/s)**	**Apical to Basolateral MDR1-MDCK (10** ^**−6**^ **cm/s)**	**Basolateral to Apical MDR1-MDCK (10** ^**−6**^ **cm/s)**
Prednisone	10	2.09	2.86	0.499	51.8
Prednisone	1	2.83	3.42	0.263	43.8
Prednisolone	10	0.294	1.31	0.285	40.8
Prednisolone	1	0.912	1.84	0.675	47.0
Deflazacort	10	6.66	6.70	1.07	62.60
Deflazacort	1	12.3	11.6	1.04	62.60
21-Des-deflazacort	10	6.28	8.8	1.11	73.6
21-Des-deflazacort	1	12.4	15.5	1.08	72.8
Vamorolone	10	33.4	22.7	8.73	56.1
Vamorolone	1	33.4	25.2	7.99	54.7

### Vamorolone and prednisolone increase CNS transcripts more than deflazacort

To better understand the biodistribution and dose-response of corticosteroids with respect to gene expression, B10-mdx mice were dosed daily for 2 weeks with deflazacort (1.25, 2.5, 5, and 15 mg/kg), prednisone/prednisolone (1.2, 2.4, 5 mg/kg), vamorolone (15, 30, and 45 mg/kg), or vehicle control (See Study 1 in the Materials and Methods). To evaluate short-term changes, after 2 weeks of treatment, the mice were euthanized, and brain, muscle, and liver tissue collected for RNA-sequencing (RNA-seq) analysis to evaluate the number of changes in gene expression in the muscle, liver, and brain.

**Figure 3:**
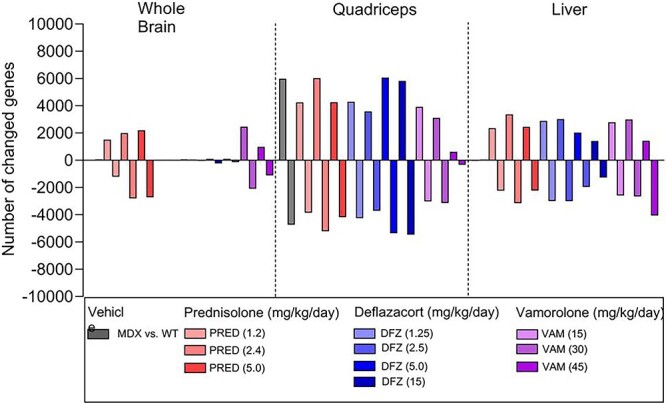
RNA-sequencing analysis of brain, quadriceps, and liver in B10-mdx mice after 2 weeks of corticosteroid treatment. Bars represent the number of genes showing greater than 2-fold change relative to vehicle in mouse whole brain, quadriceps, and liver tissues. Bars above the X-axis are up-regulated, and bars below the X-axis are down-regulated (N = 6 individual animal samples from each treatment group). See Study 1 in Materials and Methods for experimental details.

First, we compared differences in gene expression between wild-type and B10-mdx mice following 2 weeks of dosing with vehicle control. In Fig. 3, gray bars above the line indicate genes with significant increases in expression and those below are genes with decreases in expression. The greatest difference in gene expression between the wild-type and B10-mdx mice was in the muscle, with little difference in the liver and brain. (Fig. 3 and Supplementary Fig. S2 and Supplementary Table S2). This was not unexpected as the B10-mdx mouse model of DMD affects primarily the muscle tissue.

Next, we compared changes in gene expression in B10-mdx mice due to corticosteroid administration with expression in vehicle-dosed B10-mdx mice. All three corticosteroids were associated with a similar number of gene expression changes relative to vehicle in the liver and muscle (Fig. 3). However, both vamorolone and prednisolone treatment resulted in larger changes in the number of genes in the brain relative to the vehicle B10-mdx group when compared to deflazacort treatment.

### Comparison of CNS effects in prednisolone-, deflazacort-, and vamorolone-treated B10-mdx mice after 28 days of dosing

To evaluate CNS effects among the three corticosteroids, B10-mdx mice were dosed daily for 4 months (See Study 2 in Materials and Methods) with deflazacort at 1.25, 2.5, 5, and 15 mg/kg; with prednisolone at 1.2, 2.4, and 5 mg/kg; and with vamorolone at 15, 30, and 45 mg/kg, and subsequently assessed for behavioral changes.

After 4 weeks of dosing (Study 2), these mice were evaluated for corticosteroid-induced depression-like behavior. Corticosteroids have been shown to elicit depression-like behavior in rodents [33, 34]. Corticosteroid-treated mice were subjected to the force-swim test (FST) followed by the tail-suspension test (TST) 24 h later. During the TST, normal mice will struggle and constantly move. However, mice show depression-like behavior by quickly giving up, stop moving, and remaining still for a greater percentage of the time.

**Figure 4:**
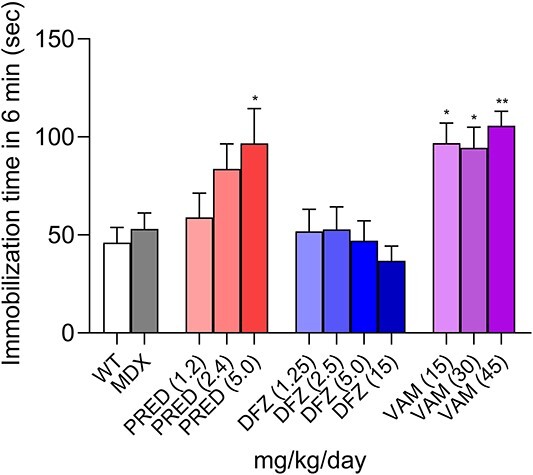
Prednisolone and vamorolone induce corticosteroid-induced depression after 28 days of treatment. Corticosteroid-induced depression as measured using the tail suspension test 24 h after the force-swim test. mdx-B10 mice were dosed for 4 weeks (see Study 2 in Materials and Methods). Bars represent the mean ± SEM total immobilization time during the 6-min tail suspension test is shown in seconds (s). Data were analyzed using a one-way ANOVA with Bartlett’s corrections and Bonferroni post hoc tests. ^*^, *P* < 0.05; ^**^, *P* < 0.01 corticosteroid-treated B10-mdx versus vehicle-treated B10-mdx. WT, N = 16; B10-mdx, N = 15; corticosteroid-treated groups, N = 8–10.

In our experiments, vehicle-treated WT and B10-mdx mice did not show significant differences in total floating time (immobilized in water) among groups in the 5-min FST (Supplementary Fig. S3a). For the TST, vehicle-treated WT control (white bar) and B10-mdx mice (gray bar) did not differ in the time that they remained still in the TST (Fig. 4), indicating that B10-mdx DMD mice are not inherently more depressed than WT mice.

Deflazacort did not increase the time the mice spent immobile relative to untreated B10-mdx mice and WT controls. The high dose of prednisolone and all doses of vamorolone increased the immobilization time significantly.

The findings described above are further supported by an additional study (see Study 3 in Materials and Methods) that evaluated behavioral effects in WT mice following daily dosing with prednisolone 15 mg/kg or vamorolone 45 mg/kg for 35 days. After 35 days of treatment, both corticosteroids induced depression-like behavior as measured by the TST (Supplementary Fig. S3b and c). In the CNS of these mice*Fkbp5* mRNA and *Ddrd4* mRNA levels were increased (Supplementary Fig. S3d and e). Expression of *FKBP5* is reported to be GRE regulated [35], and *Drd4* is a reported biomarker of depression [36, 37].

### Comparison of peripheral side effects (endogenous corticosterone, glucose, and bone parameters) in prednisolone-, deflazacort-, and vamorolone-treated B10-mdx mice

To evaluate the similarities and differences of potential side effects of the three corticosteroids, plasma/blood were evaluated ex vivo for their effects on endogenous corticosteroid after 14 days of dosing (Study 1) and glucose levels (Study 1) after 11 days of dosing. Bone formation was measured by evaluating osteocalcin levels [28, 29] in Study 1 after 14 days. Bone density was measured by micro-computed tomography (micro-CT) in Study 2 after 4 months.

**Figure 5:**
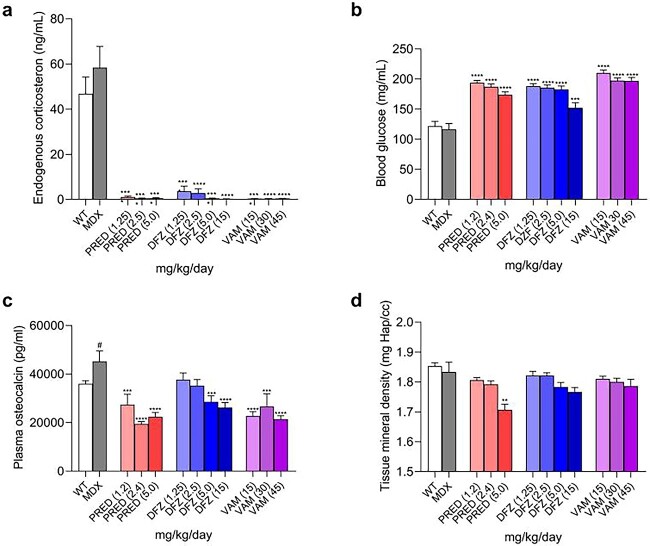
Comparison of the dose-dependent effects of corticosteroids after 11–14 days (a–c) and 4 months (d) of dosing. (a) Following 14 days of treatment with prednisolone, deflazacort, and vamorolone in B10-mdx mice, endogenous corticosteroids levels (Study 1 in Materials and Methods); (b) following 11 days of treatment, 6-h fasting blood glucose levels (Study 1 in Materials and Methods); (c) following 14 days of treatment, plasma osteocalcin levels (Study 1 in Materials and Methods; (d) following 4 months of treatment with prednisolone, deflazacort, and vamorolone in B10-mdx mice (Study 2 in Materials and Methods), femur cortical bone density shown as tissue mineral density using micro-CT imaging analysis, measured via micro-CT imaging. Data were analyzed using a one-way ANOVA with Bartlett”s corrections and Bonferroni post hoc tests. # is a Student’s t-test comparing wild-type vs mdx, *P* < 0.05; ^*^, *P* < 0.05; ^**^, *P* < 0.01; ^***^, *P* < 0.001; ^****^, *P* < 0.0001 corticosteroid-treated B10-mdx versus vehicle-treated B10-mdx. a–c, N = 10–12; d, N = 6–8.

At all doses tested in Study 1 (11–14 days), the three drugs severely reduced the levels of endogenous corticosterone (the primary mouse endogenous corticosteroid), increased glucose levels, and reduced osteocalcin levels (Fig. 5a–c).

To assess the impact of the three corticosteroids on bone density, micro-CT was used to evaluate tissue mineral density in B10-mdx mice treated with corticosteroids for 4 months. After 4 months of treatment, no significant differences were seen between WT and B10-mdx mice treated with vehicle control (white vs gray bar). Only the highest dose of prednisolone significantly reduced bone density in the femur (Fig. 5d).

### Comparison of efficacy of prednisolone, deflazacort, and vamorolone

#### Prednisolone, deflazacort, and vamorolone alter the neutrophil-to-lymphocyte ratio, cytokines, and CK after 2 weeks of treatment

**Figure 6:**
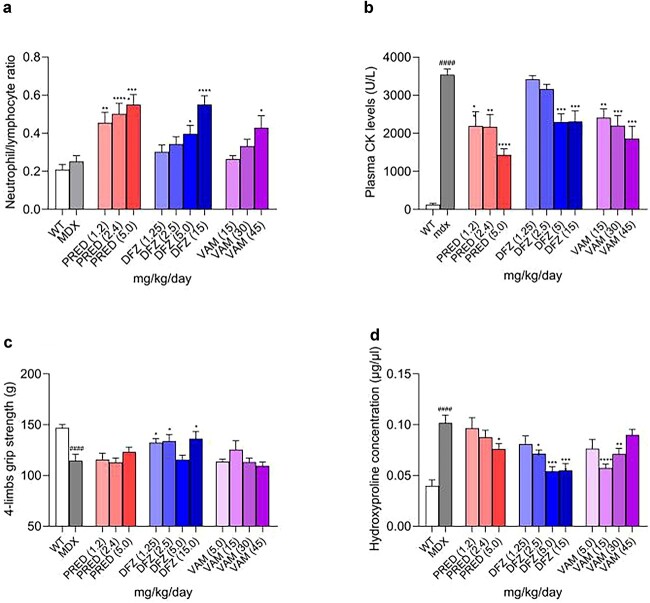
Dose-dependent effects of corticosteroids: (a) after 2 weeks of dosing: ratio of circulating neutrophils to lymphocytes, (c) after 4 weeks dosing: Grip strength, (c) after 4 months of dosing: diaphragm hydroxyproline concentration (a) numbers of circulating neutrophils and lymphocytes were measured, and the ratios are shown after two weeks of treatment (Study 1 1 in Materials and Methods). (b) CK measured after two weeks of treatment (Study 1 in Materials and Methods). (c) Four-limb grip strength was measured after 4 weeks of treatment (Study 2 1 in Materials and Methods). (c) Hydroxyproline concentration in diaphragms after 4 months of treatment (Study 2 1 in Materials and Methods). Data were analyzed by a one-way ANOVA with Bartlett’s corrections and Bonferroni post hoc tests ^*^, *P* < 0.05; ^**^, *P* < 0.01; ^****^, *P* < 0.0001 corticosteroid-treated B10-mdx versus vehicle-treated B10-mdx. ####, *P* < 0.0001 B10-mdx versus WT. N = 10–12.

It is understood that part of the efficacy of corticosteroids is via their impact on leukocyte trafficking, resulting in a decrease in lymphocyte and an increase in neutrophil counts in the blood within hours of drug administration [38, 39]. Corticostetoids regulate leukocyte trafficking via changes in adhesion molecules and cytokines, which likely reflects inhibition of NF-κB [39, 40]. The effect of the three corticosteroids on leukocyte trafficking was assessed in B10-mdx mice after 2 weeks of dosing (Study 1). No significant differences in neutrophil-to-lymphocyte ratio were seen between wildtype and B10-mdx mice (Fig. 6a).

Consistent to what is generally observed clinically [38], all three corticosteroids induced an increase in the neutrophil-to-lymphocyte ratio (increased neutrophils and decreased lymphocytes) (Fig. 6a) and were significantly greater than those observed in vehicle-dosed WT and B10-mdx mice. Furthermore, as shown in Supplementary Fig. S4 and Fig. 6b, cytokines and CK were elevated in B10-mdx mice relative to wildtype mice. Two weeks of treatment with any of the three corticosteroids decreased cytokines and CK. These data suggest all three corticosteroids inhibit NF-κB and reduce disease in the B10-mdx mouse.

#### Effect of corticosteroids on muscle

After 4 weeks of treatment, muscle strength was evaluated using a grip strength meter. Although the B10-mdx mouse has a mild phenotype relative to human disease, a statistically significant loss in grip strength was observed in the vehicle-dosed B10-mdx compared to WT mice. Of the three corticosteroids, only deflazacort showed some protection from loss of grip strength, although the response was not complete and was not dose dependent (Fig. 6c).

To determine muscle damage in B10-mdx mice and to evaluate the effects of the three corticosteroids on muscle, we used the hydroxyproline assay to measure the collagen content in diaphragm muscle tissue, a measure of muscle damage and fibrosis [41, 42]. Hydroxyproline concentration in untreated B10-mdx mice was significantly higher than that in the WT control animals, indicating severe muscle damage and subsequent fibrosis in the B10-mdx mice. Levels were reduced in mice treated with any of the three corticosteroids (Fig. 6d). Higher doses of deflazacort at 5 and 15 mg/kg and of prednisolone at 5 mg/reduced fibrosis in B10-mdx mice. Vamorolone reduced fibrosis at 15 and 30 mg/kg, but reduction did not reach significance at 45 mg/kg (Fig. 6d).

#### Prednisolone, deflazacort, and vamorolone inhibit NF-κB-driven cytokine and GRE-mediated STAT1 expression in cultured mouse macrophages

The immunosuppressive activity of corticosteroids is in part due to their inhibition of NF-κB-induced cytokines including TNF-α, IL-6, and IL-12. Furthermore, expression of some inflammatory-mediated proteins such as STAT1 is mediated via GREs, and not the NF-κB pathway [43]. We therefore expanded murine bone marrow-derived macrophages (BMDM) in vitro, and after 5 days of culture tested the relative efficacy of different corticosteroids to prevent LPS-induced cytokine secretion.

All three corticosteroids significantly decreased several proinflammatory cytokines produced by cultured BMDM cells (Table 3, Supplementary Fig. S5). BMDMs treated with corticosteroids produced reduced levels of cytokines regulated via the NF-κB pathway (i.e. IFN-γ, IL-10, Il-1β, and IL-12). Corticosteroid treatment also reduced the STAT1 transcription factor, which is regulated through the GRE pathway. The ratio of NF-κB- versus GRE-mediated expression was similar (Table 3), indicating the three corticosteroids affected these two pathways similarly.

**Table 3:** Effects of prednisolone, deflazacort, and vamorolone on levels of proinflammatory cytokines.

nM	Deflazacort EC_50_	Prednisolone EC_50_	Vamorolone EC_50_
IFN-γ	0.29	0.50	0.8
IL-1β	1.3	3.0	32
IL-12	3.6	8.5	30
STAT1	3.1	9.4	7.6
STAT1/IFN-γ Ratio	11×	19×	10×

n.d., not determined. EC_50_ for cytokine inhibition for corticosteroid treated BMDM culture cells with different drug concentrations were measured and shown as nM. Graphs representing these data are presented in the Supplementary Fig. S6.

## Discussion

This study was designed to evaluate the dose-dependent efficacy and safety profile of the corticosteroids prednisone/prednisolone, deflazacort, and vamorolone in the B10-mdx mouse model of DMD. All three corticosteroids showed side effects at doses associated with efficacy in the B10-mdx mouse model.

DMD is associated with behavioral challenges, and corticosteroids can exacerbate these challenges. In this study, CNS drug accumulation, changes in brain gene expression, and behavior in B10-mdx mice treated with prednisolone, deflazacort, or vamorolone were measured. The study found that vamorolone accumulated in the brain after oral dosing and that the brain/plasma ratio was higher than that of prednisolone and deflazacort. The accumulation of vamorolone in the brain may, at least in part, reflect the fact that vamorolone is a poor substrate for P-gp, a protein that pumps drugs out of the CNS. Deflazacort showed the least number of changes in gene expression in the CNS.

Doses of vamorolone used in the current studies were based on doses previously reported as effective in the mdx mouse model. The dose of vamorolone required to see efficacy in the mouse results in a higher exposure than does the exposure associated with efficacy in the clinic [23, 24]. Specifically, the dosing of vamorolone at 15, 30, and 45 mg/kg resulted in 2–4-fold higher exposure in the mouse (based on C_max_) than the human clinical exposure following a 6 mg/kg/day dosing (Supplementary Table S3). A dose of 4 to 5 mg/kg would be predicted to have similar exposure in the mouse as clinical 6 mg/kg/day dose. However, published data reported that administration of vamorolone at 5 mg/kg in the mdx mouse showed little activity [24]. Similarly, in our studies, we also saw limited activity at 15 mg/kg and hence did not include lower doses in our experiments that would more closely match the PK AUC of the 6 mg/kg clinical dose. At a dose of 15 mg/kg vamorolone, limited biological activity was noted and defined by the decrease in lymphocytes or the increase in neutrophils after 2 weeks of dosing (Supplementary Table S4). For deflazacort and prednisolone/prednisone, doses of approximately 5 mg/kg in the mouse resulted in similar exposures as the human clinical dose of 0.9 and 0.75 mg/kg, respectively (Supplementary Table S3). The dose of prednisolone in these studies used may have resulted in greater biological activity systemically in the mouse, as the lowest dose of prednisolone resulted in similar changes in the neutrophil-to-lymphocyte ratio as the highest doses of deflazacort and vamorolone. The difference between vamorolone exposures associated with efficacy in mice and humans may reflect differences in the PK profile (shape of the PK curve) or differences in binding of the corticosteroid to the mouse compared with the human glucocorticoid receptor. These differences may influence the efficacy/side effect ratio across species making direct translation from the mouse to the human difficult.

A key finding in these studies is that vamorolone is not pumped out from the CNS as effectively as is deflazacort or prednisolone so vamorolone accumulates in the CNS in the mouse. This may contribute to gene expression changes in the CNS and changes in behavior. Deflazacort and prednisolone are strong P-gp substrates and were found at low levels in the CNS of the mouse. Our results are consistent with published data showing that both deflazacort and its active metabolite are P-gp substrates (package insert) as are prednisolone/prednisone [44]. Limited data are published on the biodistribution of corticosteroids, but the ratio of prednisolone in the CSF to the plasma after IV administration to the non-human primate was reported as 0.08 [45] and was reported to be lower in wildtype mice than in P-gp knockout mice [44]. RNA-seq analysis found that prednisolone and vamorolone were associated with a greater number of changes in gene expression in the brain compared with deflazacort. This may suggest that the levels of prednisolone were still sufficiently high to drive gene transcription, possibly because the biological activity of prednisolone at the highest dose as defined by the neutrophil-to-lymphocyte ratio (the lowest dose of prednisolone was equivalent to the highest dose of vamorolone).

The effect of the three corticosteroids on behavior was assessed using the tail-suspension test, a mouse model of corticosteroid-induced depression-like behavior [34]. Vamorolone and prednisolone effectively induced depression-like behavior, consistent with the RNAseq expression data. Our results with vamorolone differ from a recent publication assessing efficacy of vamorolone from the laboratory of Heier *et al.* [46]. In that study using a Becker muscular dystrophy mouse model, they found that prednisolone at 5 mg/kg elicited a behavioral change (increased anxiety measured in the elevated plus maze), whereas in contrast to the effect on depression-like behavior published here, vamorolone at 20 mg/kg did not induce a behavioral change.

DMD is often associated with behavioral and cognitive abnormalities as dystrophin has a biological function in the brain [47, 48]. However, there is no evidence that dystrophin deficiency in the brain causes inflammation as is seen with skeletal muscle. Therefore, a lower brain/muscle ratio of anti-inflammatory drugs is likely preferable for DMD. However, or other indications, such as the use of corticosteroids for treatment of brain tumors, vamorolone may be preferable [49].

One known side-effect of corticosteroids is suppression of endogenous corticosteroid production. Our results regarding endogenous corticosteroid suppression align with the published data. We demonstrated that all three corticosteroids reduce endogenous corticosteroid production in vivo. While it has been hypothesized that vamorolone does not reduce endogenous corticosteroid production, published data have shown that both prednisolone/prednisone and vamorolone showed concentrations-dependent inhibition of ACTH secretion in a cultured pituitary cell line. Although vamorolone showed less reduction in ACTH secretion compared with prednisolone/prednisone [24], it is unclear how this difference in vitro correlates quantitatively to endogenous corticosteroid production in vivo. Further, inhibition of endogenous corticosteroids was corroborated in a Phase 2a study where treatment with vamorolone at 6 mg/kg/day for 2 weeks resulted in adrenal suppression [27]. Similarly, in a randomized double-blind 24-week study in boys with DMD (N = 133) vamorolone (dosed 2 mg//kg/day or 6 mg/kg/day) and prednisolone/prednisone (0.75 mg/kg/day) significantly reduced morning cortisol levels at both 12- and 24-weeks from baseline, with the 2 mg/kg/day showing significantly less reduction in adrenal suppression than prednisone (*P* < 0.001) [30]. Vamorolone administered at 6 mg/kg/day showed significantly greater adrenal suppression than prednisolone/prednisone (*P* = 0.03) in a trial including DMD boys aged 4–7, but was also equal or more efficacious in preventing DMD-associated loss of muscle function [30].

Another side-effect of corticosteroids is alterations in endocrine function including regulation of glucose metabolism. Our studies showed that all 3 corticosteroids resulted in an increase in fasting glucose levels, with no differentiation between the three at the doses tested. We did not look at insulin levels. Preclinical studies evaluating the effect of vamorolone on glucose have not been reported, although Heier *et al.* saw no increase in insulin in mdx mice with dosing of 30 mg/kg of vamorolone under conditions where 5 mg/kg of prednisolone elicited an increase in insulin levels [23]. Similarly, a clinical study indicated vamorolone is associated with less insulin resistance than is prednisolone/prednisone [27]. No preclinical or clinical studies have reported fasting glucose levels.

Another side-effect of corticosteroids is osteoporosis/osteopenia. In these studies, the three corticosteroids significantly reduced osteocalcin serum levels after 2-weeks of treatment, a biomarker of bone formation. Further, prednisolone/prednisone significantly reduced bone density measured by microCT after 4 months of treatment, with the differences induced by deflazacort and vamorolone not reaching significance. This is consistent with the Phase 2B study comparing vamorolone with prednisolone, in which vamorolone induced less stunting of growth than did prednisolone [30].

These results are consistent with clinical data assessing vamorolone after 2 weeks of treatment, although not with longer vamorolone clinical use. Consistent with the preclinical studies, findings from a Phase 2A study in subjects with DMD (N = 48) indicated that in humans vamorolone at 6 mg/kg/day reduced serum osteocalcin levels after 14 days of dosing by ~33% [27]. The reduction of osteocalcin levels from baseline with the lower 2 mg/kg/day dose tested was not significant [27]. In a Phase 2b 24-week study in DMD boys found osteocalcin levels were minimally reduced with 6 mg/kg vamorolone treatment (0.17%) and significantly reduced (15%) with 0.75 mg/kg prednisone. Similarly, an open-label, multiple ascending dose study in boys with DMD (N = 48) also found no significant change was observed with the 6.0 mg/kg/day dose [26]. Potentially, vamorolone may have a better bone safety profile than prednisolone/prednisone [27].

Corticosteroids provide benefit in patients with DMD by reducing inflammation. Most cytokines are regulated by NF-κB, but some other factors are regulated by GRE sites. The three corticosteroids all reduced proinflammatory cytokines produced by cultured primary bone-derived mouse macrophages (i.e. IFN-γ, IL-10, Il-1β, and IL-12) via the NF-κB-dependent pathway, but all three corticosteroids also induced protein regulated by directly binding GREs (i.e. STAT expression).

Corticosteroids impact leukocyte trafficking, causing increases in the levels of neutrophils and decreases in the levels of lymphocytes [38, 39]. Corticosteroids regulate leukocyte migration via changes in adhesion molecules and cytokines, which likely reflects inhibition of NF-κB [39, 40]. Consistent with this known action of these drugs, all three corticosteroids induced an increase in neutrophils and a decrease in lymphocytes.

All three corticosteroids showed efficacy as demonstrated by reductions in CK and cytokines. In a test of muscle strength, improvement with deflazacortrelative to vehicle reached statistical significance. In addition, differences in fibrosis in mice dosed with deflazacort and prednisolone relative to vehicle reached statistical significance.

No clinical studies have been done comparing these 3 corticosteroids side-by-side. Post-hoc evaluation of DMD trials has suggested that long-term treatment with deflazacort is more effective than prednisolone/prednisone [13, 50–52]. However, the recent trial, FOR DMD, compared deflazacort and prednisone and found no significant differences between prednisolone and deflazacort in young DMD patients who were 4 to 7 years of age [53]. The FOR-DMD study was designed to evaluate the initial treatment effects in these young boys and not the later efficacy of these corticosteroids [53].

In conclusion, the findings described here indicate that prednisone/prednisolone, deflazacort, and vamorolone have similar safety profiles in the B10-mdx DMD mouse model and are consistent with the known safety and molecular effects of corticosteroids in the clinic. Possibly, the differences between these corticosteroids can be exploited to best treat specific diseases or patients. However, given the limitations in comparing these compounds in preclinical studies, additional work would be needed to better translate these findings to the clinic.

## Materials and Methods

### Materials

Vamorolone was synthesized using published methods [54]. Prednisolone was purchased from Sigma Aldrich, P6004 (Clinical grade). Deflazacort (Emflaza™) was provided by PTC Therapeutics.

### MDCK-MDR1 permeability assay

Testing for permeability and MDR1 substrate assessment was done in MDCK cells transfected to overexpress human MDR1 gene that encodes efflux transporter protein (P-gp) [55]. Cells that do not express human MDR1 were used as a control. Briefly, cells were maintained in Dulbecco’s modified Eagle medium (DMEM) with 10% fetal bovine serum and 1% penicillin/streptomycin in a humidified incubator at 37°C with 5% CO_2_. The study was performed by Absorption Systems (now Pharmaron; Exton, PA). The MDR1-MDCK and MDCK control cell lines were seeded into 12-well plates for 7 days. Integrity of monolayer tight junctions was monitored preincubation by transepithelial electrical resistance (TEER) measurement. Aliquots of prednisolone, prednisone, or vamorolone in DMSO stock were added to Hanks’ balanced salt solution (HBSS) containing 4-(2-hydroxyethyl)-1-piperazineethanesulfonic acid (HEPES) and 15 mM glucose, at pH 7.4; the nominal test concentrations were 1 and 10 μM, and the compounds were tested in duplicate. The samples were withdrawn at t = 0 and 60 min from the receiver and donor compartments. Compound levels were measured by LC/MS–MS. Additionally, post-assay, cell monolayer integrity was checked with Lucifer Yellow permeability measurement. Transport assay low- and high-permeability control compounds were run in parallel for assay validation. P_app_ values were calculated considering compound concentrations across the monolayer and cell surface area. Efflux ratios were calculated based on P_app_ B > A over P_app_ A > B. The difference between the MDR1-MDCK and the MDCK cell efflux ratios was also calculated. Control compounds were included: atenolol (P_app_ 0.0464–0.0873 10^−6^ cm/s), propranolol (P_app_ 19.7–21.4 10^−6^ cm/s), and digoxin (A®B P_app_ 0.0521–0.0558 10^−6^ cm/s; B®A P_app_ 19.6–20.7 10^−6^ cm/s; efflux ratio 352–398). Compounds were classified by Absorption Systems/Pharmaron as follows:

*Brain Penetration Potential Classification:*

Papp (A-to-B) ≥ 3.0 and ER < 3.0: **High.**

Papp (A-to-B) ≥ 3.0 and 10 > ER ≥ 3.0: **Moderate.**

Papp (A-to-B) ≥ 3.0 and ER ≥ 10, or Papp (A-to-B) < 3.0: **Low.**

### Animal studies

All animal studies were performed under IACUC-approved protocols at AAALAC-certified animal facilities. All functional/behavioral experiments were performed by scientists blinded to genotype and treatment groups. Animals were housed in a controlled vivarium with a 12-h light/dark photoperiod. Animals had free access to food and water.

#### Pharmacokinetic studies in animals

Pharmacokinetics (PK) of vamorolone, deflazacort, and prednisolone was evaluated in WT DBA/2 J mice. Deflazacort (4 mg/kg) and prednisolone (5 mg/kg) were formulated as a suspension in 0.1% hydroxypropyl methyl cellulose (HPMC) with 0.5% Tween 80 (at pH 4.0) and administered by gavage at 10 ml/kg. Vamorolone (5, 15, 30, or 45 mg/kg) was formulated in cherry syrup (Fisher Scientific) and administered by gavage at 5 ml/kg. Blood was collected by terminal cardiac puncture at specified time points (three mice per time point; 5 min, 15 min, 30 min, 1 h, 1.5 h, 2 h, 4 h, 7 h, and 24 h) and centrifuged to collect plasma. Brain tissue was collected at the time of blood collection and homogenized in saline. The concentrations of test compounds in plasma and brain tissue were quantified by LC-MS/MS. Briefly, plasma and homogenized tissue samples were treated with acetonitrile-methanol mixture containing an internal standard that is a close structural analog of the test compounds. The treated plasma and homogenized brain samples were centrifuged, and the supernatant was collected and analyzed using electrospray LC-MS/MS. The lower limit of quantification (LLOQ) of 21-des-deflazacort (the active metabolite of deflazacort), vamorolone, and prednisolone were 1 ng/ml in the plasma and 5 ng/mg wet tissue in the brain. The area under the concentration versus time curve was calculated for both the plasma and the brain concentration curves using Phoenix WinNonLin v8 (Certara, Princeton, NJ).

#### In vivo functional studies

Three studies were designed and performed as outlined in Fig. 2. Shown is the dose (mg/kg) and the number of animals at that dose level. For the first two studies, *C57BL/10ScSn-Dmd^mdx^/J* (*B10-mdx*) 6-week-old male heterozygous mice (stock #001801) that carry a loss-of-function mutation in the dystrophin (*Dmd*) gene and their age-matched WT male mice, *C57BL/10ScSnJ* (*B10-WT*) (stock #000476), were purchased from The Jackson Laboratory (Bar Harbor, Maine). For the third study, male C57BL/6 J were utilized and purchased from The Jackson Laboratory, stock #000664).

**Study 1:** The goal of this study was to define the dose-response of each of the three corticosteroids. B10-mdx mice were dosed daily by oral gavage for 14 days with deflazacort (1.25, 2.5, 5, and 15 mg/kg), prednisone/prednisolone (1.2, 2.4, 5 mg/kg), or vamorolone (15, 30, and 45 mg/kg). On Day 11, mice were fasted for 6 h and then blood was obtained to measure blood glucose. Blood glucose was measured using a glucometer (ACCU-CHECK) by tail bleeding. At the end of 14 days, mice were euthanized, and blood samples were evaluated for complete blood count (CBC) and plasma were collected for analysis of corticosterone levels, osteocalcin, creatine kinase, and cytokines. Brain, muscle, and liver were collected for RNAseq. The number of animals per group was as follows: WT, n = 15; B10-mdx, n = 11; corticosteroid-treated groups, n = 10–12.

**Study 2:** The goal of the second study was to evaluate corticosteroid side effects and efficacy in a longer study (4 months). After 28 days of dosing, mice were tested for depression-like behavior using a measure of corticosteroid-induced depression (FST followed by TST). Muscle function was tested via grip strength. Tests for depression and for grip-strength were done in a blinded manner. After 4 months, mice were euthanized, and tissues were collected. Bone from whole left leg was collected for micro-CT analysis. The number of animals per group was as follows: WT, n = 16; B10-mdx, n = 15; corticosteroid-treated groups, n = 8–10.

**Study 3:** The goal of the third study (35 day) was to confirm that prednisolone and vamorolone induce corticosteroid-induced depression-like behavior in WT mice and to allow for collection of brain tissues to measure genes associated with depression.

#### Endpoints and ex vivo analysis

*Corticosterone*

Corticosterone was measured using the Parameter™ Corticosterone ELISA assay (R&D Systems, cat #KGE009), which is based on the competitive binding technique. A total of 50 μl plasma was used and diluted 1:4 in the final assay.

*Osteocalcin*

Osteocalcin was measured using a mouse osteocalcin ELISA kit (LifeSpan Biosciences, cat #LS-F12227). A total of 5 μl plasma was used and diluted 1:50 in the final assay.

*Cytokine*

Plasma cytokine levels were measured using Milliplex Mouse, a Cytokine/Chemokine Magnetic Bead Panel 96-well assay from Millipore-Sigma (cat #MCYTOMAG-70 K). A total of 35 μl plasma was used and diluted 1:2 in the final assay.

*Hydroxyproline Assay*

Diaphragm muscles were dissected, weighed, and stored at −80°. Tissues were cut to avoid the tendon(s), and 10 ± 1 mg of tissue was homogenized through sonication. Water and hydrochloric acid (12 M) content were normalized prior to hydrolysis at 120° for 3 h in pressure-tight polypropylene vials. After boiling, the hydrolysates were centrifuged at 10 000 × *g* for 3 min, and 6-μl triplicates of the hydrolysate were added to clear 96-well plates for evaporation in a 60°C oven overnight. The following day, a hydroxyproline kit (Sigma, cat #MAK008) was used to determine the hydroxyproline content. In each sample and standard well, 100 μl Chloramine T/Oxidation buffer mixture was added, incubated for 5 min at room temperature, and followed with 100 μl of perchloric acid diluted DMAB concentrate. The plate was sealed and incubated in a 60°C oven for 90 min. The 96-well plates were read using a BioTek Epoch 2 Spectrophotometer at an absorbance of 560 nm.

*Blood Glucose Measurement*

To determine blood glucose level in mdx-B10 mice after 11 days of dosing, food was removed (water was available) for 6 h prior to measurement. After 6 h of fasting, a blood sample was obtained via the tip of the tail from each mouse. Blood glucose was measured using a blood glucose monitoring system (OneTouch Verio Reflect, Lifescan, Switzerland).

*Grip Strength*

A four-limb grip strength meter from Bioseb was used to determine the maximal peak force (g) developed by the mouse when the examiner tried to pull it out of a specially designed grid for mice (Bioseb, BIO-GS3 & BIO-GRIPGS). The examiner was blinded, and a total of 5 measurements were applied to each mouse, 1–2 min between measurements. The average of the 5 values was used for subsequent calculations.

#### Corticosteroid-induced depression

In rodents, repeated administration of endogenous or synthetic glucocorticoids induces depression-like behaviors [33, 34]. Our protocol was based on data from Zhao 2008 showing an effect on the TST conducted 24 h after the FST. They reported no change in the FST but decreased mobility measured using the TST.

1) Force-swim test: Animals were single housed and acclimated in the behavior room for 30 min prior to test. A clean 1-L beaker was filled with 700 to 800 ml of 23°C water. Each mouse was placed into the water for 6 min. The total immobility time was recorded. After the test, the animals were dried and placed in a cage on a heating pad for about 10 min before returning to the home cages.
2) Tail-suspension test: Isolated mice were suspended 50 cm above the floor by surgical tape placed approximately 1 cm from the tip of the tail for 6 min. The total immobility time of mice was measured. Mice were considered immobile only when they hung passively and completely motionless.

*Micro-computed tomography (Micro-CT)*

Micro-CT analysis was performed by the *Rutgers University Molecular Imaging Center (RUMIC)*. Briefly, skeletons were harvested at trial endpoint and stored in 70% ethanol and transferred for imaging analysis. Hindlimbs were soaked in isotonic saline solution overnight to rehydrate before imaging. Each hindlimb was placed in a 5-mm-diameter plastic tube mounted in the Skyscan 1272 scanner. Samples were scanned at 6-μm image pixel size, 1092 × 1632 pixels/image, and 0.5-mm Aluminum filter. The rotation step was 0.4° through 180° total rotation, and three images were averaged at each rotation step. Each distal femur was scanned separately, and images were saved. Scan images were reconstructed using Skyscan nRecon software.

*RNA-sequencing analysis*

To identify the genomic information and variations in B10-mdx mice compared to their WT controls, whole transcriptome RNA-seq was applied. After 14 days of corticosteroid treatments, B10-mdx mice and their WT controls were euthanized by CO_2_. The whole brain, quadriceps, and liver samples were taken and frozen in liquid nitrogen and transported to Admera Health for RNA-seq analysis (Admera Health, Genomics-and-Bioinformatics, RNA-seq). Raw RNA-seq reads were mapped to mouse genome (mm9) using STAR (version 2.7) [56]; only uniquely mapped reads (with mapping quality score or MAPQ > 10) with <5% mismatches and properly paired reads were used. For gene expression analysis, the number of reads in the coding sequence (CDS) region of protein-coding genes and exonic region of noncoding genes were counted and analyzed using DESeq2 [57]. The thresholds for differentially expressed genes (DEGs) are 1) absolute value of fold change >2, 2) DESeq2 adjusted *P* < 0.05, and 3) higher one of the gene expressions measured by fragments per kilobase of transcript per million fragments mapped (FPKM) >1. The code for the RNA-seq data analysis can be found in GitHub (https://github.com/liwc01/DEDSeq).

*RT-qPCR*

Total RNA was isolated from approximately 100 mg of whole-brain tissues and quadriceps using a QIAcube HT (Qiagen, cat #9001793) with an RNeasy 96 QIAcube HT kit (Qiagen, cat #74171), QIAzol Lysis Reagent (Qiagen, cat #79306), chloroform (Fisher, cat #C606-1), absolute ethanol (Fisher, cat #A409-4), and RNase-free DNase set (Qiagen, cat #79254). RNA concentration was measured by using Cytation 5 (BioTek). For the RT-qPCR system: master mix: AgPath-ID™ One-Step RT-PCR Reagents (Thermo Fisher, cat #4387391); probes: *Fkbp5* TaqMan Gene Expression Assay, mouse, FAM-MGB (Thermo Fisher, cat #4351372, Assay Id Mm00487403_m1). *Drd4* TaqMan Gene Expression Assay, mouse, FAM-MGB (Thermo Fisher, cat #4351372, Assay Id Mm00432894_g1). *GAPDH* TaqMan Gene Expression Assay, mouse, VIC-MGB (Thermo Fisher, cat #4448490 [PN4448-491], Assay Id Mm99999915_g1). RT-qPCR reactions were as follows: 45°C 10 min, 95°C 10 min, 95°C 15 s, 60°C 45 s × 40 cycles using a QuantStudio 7 Pro (Thermo Fisher), and were assembled automatically using a QIAgility (Qiagen, cat #9001731).

*Mouse BMDM cultures*

BMDM mouse bone marrow derived macrophage (mBMDM) cultures were generated from the femur and tibia from two 7–11-week-old C57BL/6 mice. Briefly, bone marrow cells were carefully flushed out, suspended in ice-cold PBS, and spun down. After the supernatant was removed, the pelleted cells were resuspended in 2 ml (ammonium-Chloride-Potassium) ACK lysis buffer (Thermo Fisher, Cat# A1049201) and incubated for 3–4 min, followed by 14 ml ice-cold PBS, and then spun down. The pelleted cells were resuspended in 43 ml complete media [Roswell Park Memorial Institute (RPMI 1640), Gibco, Cat# 11875-093; 10% heat-inactivated fetal bovine serum (HiFBS), HyClone, Cat# SH30406.02; 1% 2 mM L-glutamine; 1% penicillin-streptomycin, Sigma, Cat# P4333-100ML; 1% MEM NEAA, Gibco, Cat# 11140-050; 1% Na Pyruvate, Gibco, Cat# 11360-070; 55 μM 2-Mercaptoethanol, Gibco, Cat#21985-023]. A total of 25 ng/ml M-CSF was added to the culture. Cells were dispensed as 200 μl per well into a 96-well tissue culture plate and incubated at 37°C, 5% CO_2_ for 4 days. On Day 5, media were removed and 100 μl fresh complete media containing M-CSF were added. Corticosteroids were added and cells incubated at 37°C and 5% CO_2_ overnight. Untreated cells were incubated with media. On Day 6, 10 ng/ml of lipopolysaccharide (LPS) was added and incubated at 37°C and 5% CO_2_ for 3 h followed by removal of cell media for cytokine analysis. Cytokines were measured using a Meso Scale Discovery (MSD), V-PLEX kit (V-PLEX Cat# K-15048D, www.mesoscale.com).

The EC_50_ values representing 50% inhibition of the maximal cytokine response were calculated using a least square fit using 4-parameter in GraphPad Prism.

### Statistics

Examiners and scientists who performed FST, TST, blood glucose measurements, micro-CT, and grip strength were blinded to treatment. In the mdx mouse experiments, the studies were powered to make multiple comparisons of the actively treated groups to the mdx vehicle control and not between each treatment group.

Statistical analysis was performed with SPSS using two-tailed Student’s *t-*test, one-way ANOVA, two-way ANOVA with Bartlett’s test corrections, and Bonferroni post hoc tests with the details included in figure legends. P values < 0.05 were considered statistically significant. The number of samples/replicates (N) are reported in the figure legends.

## Supplementary Material

SUPPLEMENTARY_DATA_FOR_RESUBMISSION_AUG2023_ddad173
