# Supplementary material for: Comparison of pharmaceutical properties and biological activities of prednisolone, deflazacort, and vamorolone in DMD disease models
Source: Hum Mol Genet. 2023 Oct 11;33(3):211–23. doi: 10.1093/hmg/ddad173 (PMC10800023; doi:10.1093/hmg/ddad173)
Supplement: SUPPLEMENTARY_DATA_FOR_RESUBMISSION_AUG2023_ddad173 [file supplementary_data_for_resubmission_aug2023_ddad173.docx]

**SUPPLEMENTARY DATA**

**Supplementary Figure S1**

**Pharmacokinetics in C57BL/10 Mice: Plasma Concentrations**


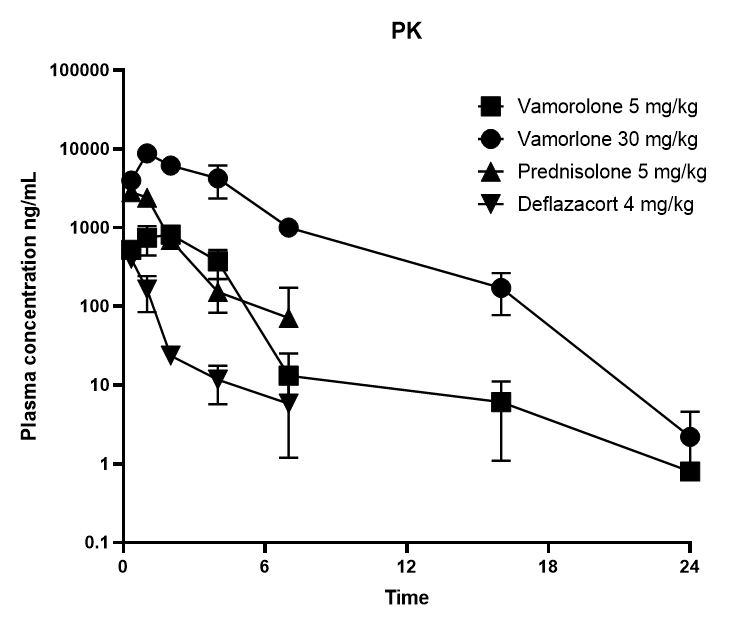


**Supplementary Table S1**

**Biodistribution in C57BL/10 Mice**

|  | **Prednisolone**  **(5 mg/kg)** | **Deflazacort**  **(4 mg/kg)** | **Vamorolone**  **(5 mg/kg)** | **Vamorolone**  **(30 mg/kg)** |
| --- | --- | --- | --- | --- |
| Plasma AUC (hr∙ng/mL) | 4636 | 376 | 2851 | 33937 |
| Plasma C_max_ (ng/mL) | 2831 | 402 | 818 | 8787 |
| Brain AUC (hr∙ng/g of tissue) | 166 | 18 | 1418 | 15538 |
| Brain C_max_ (ng/g of tissue) | 119 | 17 | 443 | 4402 |
| Brain/plasma ratio based on AUC | 0.036 | 0.049 | 0.50 | 0.46 |
| Brain/plasma ratio based on C_max_ | 0.042 | 0.043 | 0.54 | 0.50 |

**Supplemental Figure S2.** **Heatmap Shows Changes in Gene Expression in Brain, Quadriceps, and Liver in Different Doses of the Three Corticosteroids (Study 1, 2-Week Treatment of mdx Mice)**


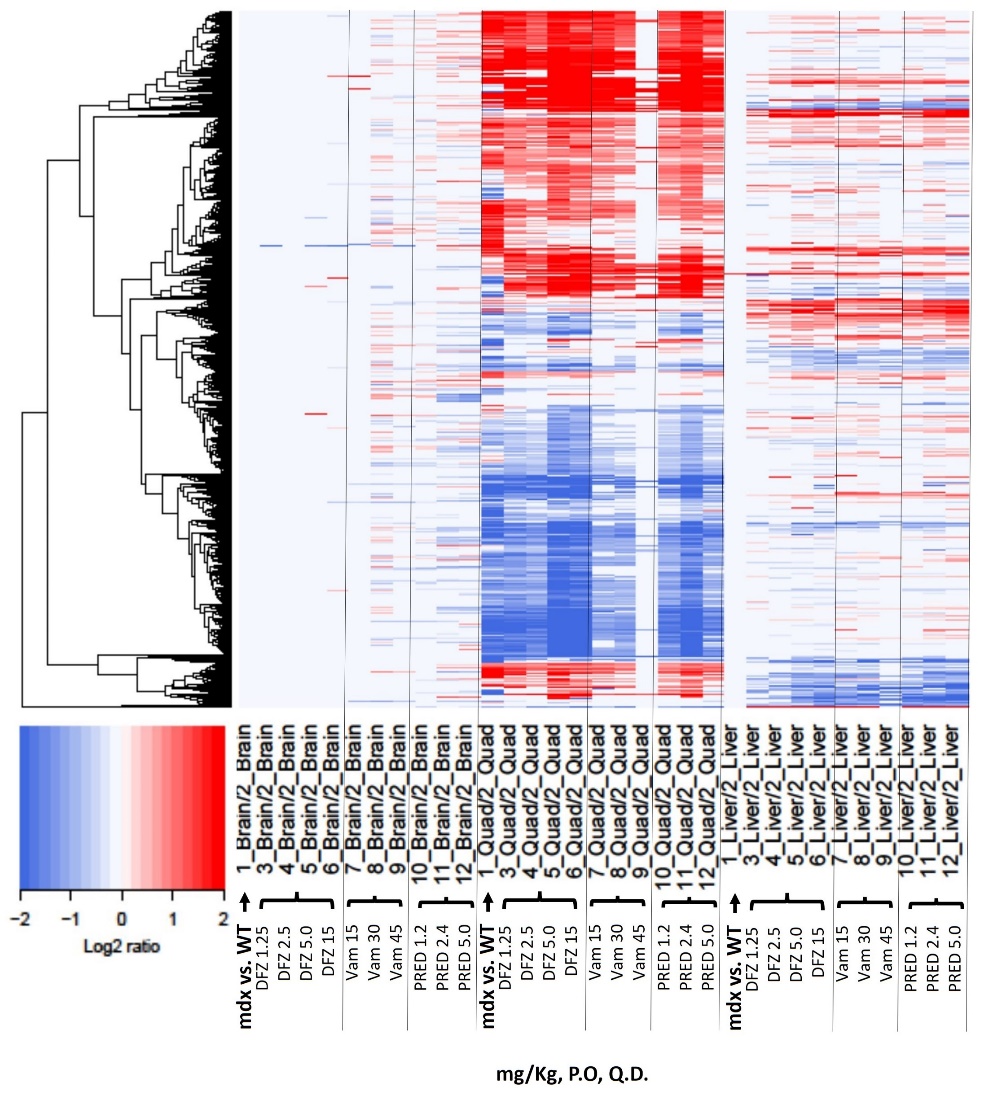


**Legend:** Heatmap shows total changes in gene expression in all three corticosteroids-treated mice whole brain, quadriceps, and liver tissue with different doses. X-axis: groups of comparisons and corticosteroid doses. Y-axis: hierarchical changes in gene expression. Red: up-regulated greater than twofold; blue: down-regulated greater than two-fold.

**Supplemental Table S2.** **Summary of the Number of Genes Whose Expression was Altered with Corticosteroid Therapy in the Brain and Quadriceps of the B10-mdx versus Vehicle-treated B10-mdx Mice (Study 1, 2-Week Treatment of mdx Mice)**

|  | **Whole Brain** | | | **Quadriceps** | | |
| --- | --- | --- | --- | --- | --- | --- |
| Treatment | Up-regulated | No Change | Down-regulated | Up-regulated | No Change | Down-regulated |
| mdx vs WT (untreated) | 0 | 16307 | 3 | 5984 | 5560 | 4730 |
| Pred 1.2 | 1511 | 13594 | 1205 | 4250 | 8205 | 3855 |
| Pred 2.4 | 1993 | 11521 | 2796 | 6030 | 8064 | 5216 |
| Pred 5.0 | 2195 | 11396 | 2719 | 4254 | 7883 | 4173 |
| DFZ 1.25 | 1 | 16287 | 31 | 4298 | 7767 | 4245 |
| DFZ 2.5 | 0 | 16306 | 4 | 3580 | 9024 | 3706 |
| DFZ 5 | 57 | 16214 | 39 | 6067 | 4889 | 5354 |
| DFZ 15 | 98 | 15981 | 231 | 5824 | 5024 | 5462 |
| Vam 15 | 104 | 16068 | 138 | 3927 | 9369 | 3014 |
| Vam 30 | 2463 | 11760 | 2087 | 3119 | 10053 | 3138 |
| Vam 45 | 978 | 14218 | 1114 | 615 | 15364 | 331 |

DFZ, deflazacort; Van, Vamorolone; Pred, prednisolone

**Supplemental Figure S3. Summary Results of Corticosteroid-induced Depression in Behavioral, Biomarker Studies in B10-mdx (Study 2) and C57BL/6 Mice (Study 3)**


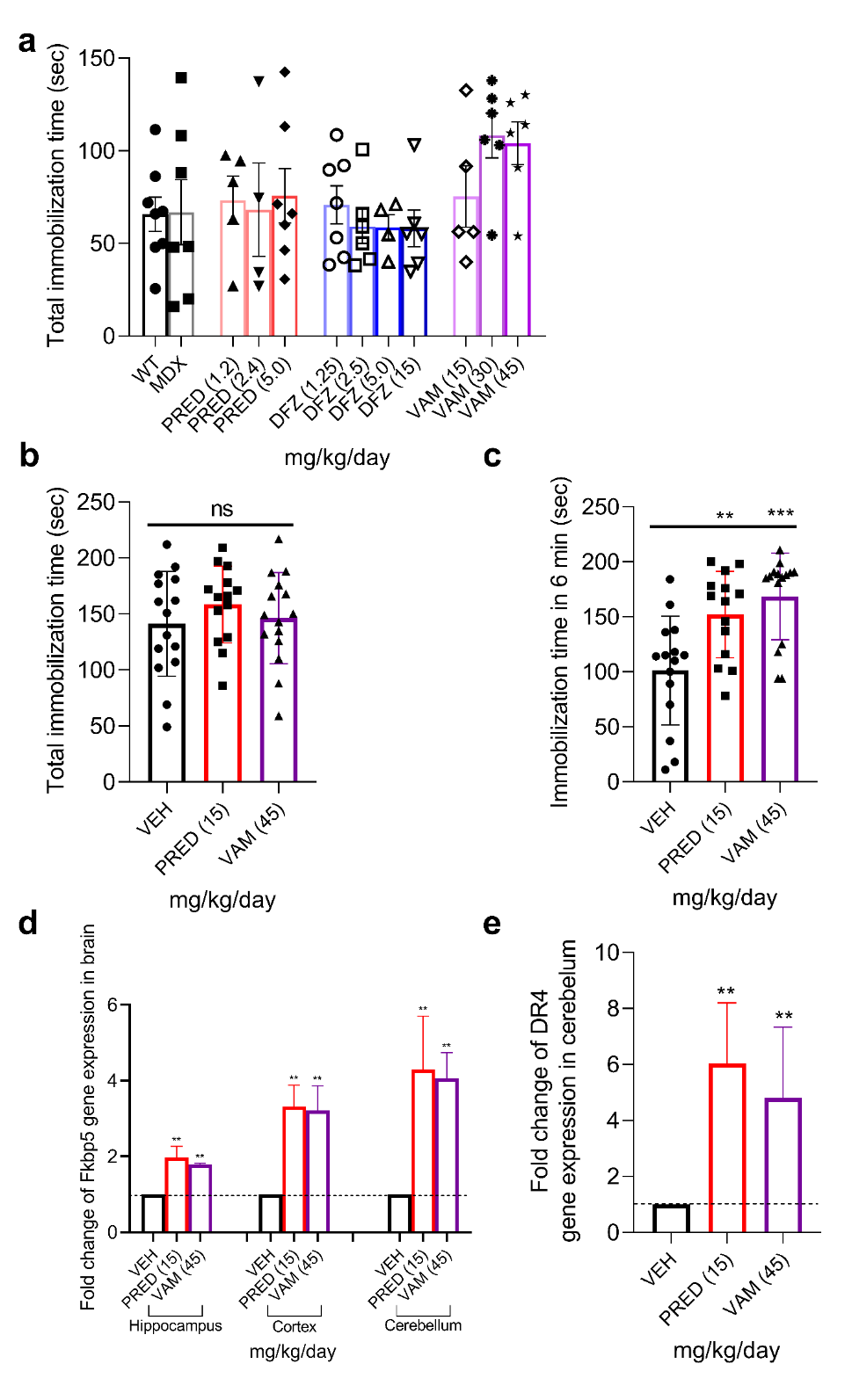


**Legend:** (a) B10-mdx mice after 4 months of corticosteroid treatment (Study 2) were subject to the forced-swim test 24 hours before tail-suspension test. Shown is the amount of time that mice remained immobile over 5 minutes. (b) C57BL/6 mice treated for 35 days with corticosteroids (Study 3) were subject to the forced swim procedure followed 24 hours later by the tail suspension test (c). *Fkbp5* (d) and *DR4* (e) mRNA expression in the brain after 35 days of corticosteroid treatment of C57BL/6 mice (Study 3). Data analyzed by ANOVA with Bartlett’s corrections and Bonferroni post hoc tests **, *p*<0.01; ***, *p*<0.001; versus WT vehicle controls. n=14 in forced swim and tail suspension tests; n=3 in RT-qPCR tests.

**Supplemental Figure S4 Plasma Cytokine Levels in Glucocorticoid-Treated mdx Mice (Study 1, 2-Week Treatment of mdx Mice)**


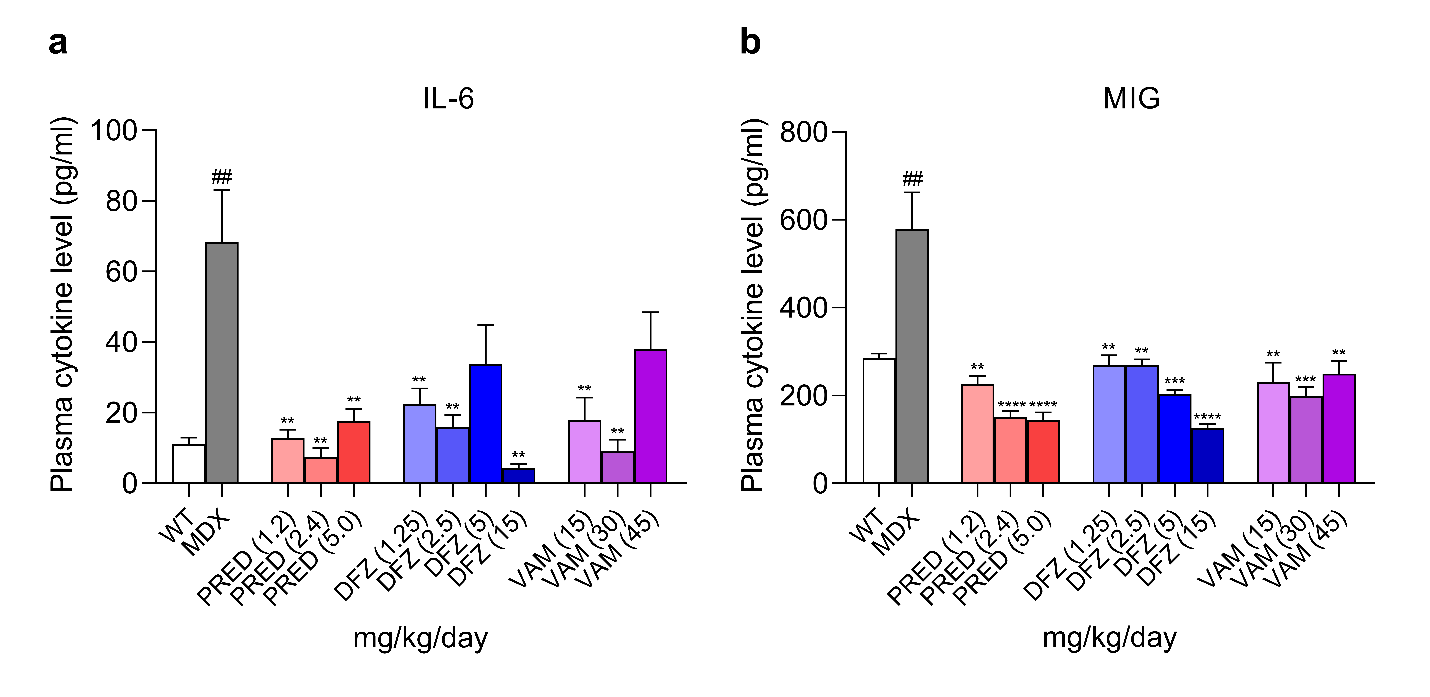


**Legend:** Plasma cytokine levels (a) IL-6 and (b) MIG. Data analyzed by one-way ANOVA with Bartlett’s corrections and Bonferroni post hoc tests. ##, *p*<0.01 versus WT vehicle controls; **, *p*<0.01; ***, *p*<0.001; ****, *p*<0.0001 versus mdx. n=10-12.

**Supplemental Figure S5 Graphic results including I_max_ and IC_50_ values**


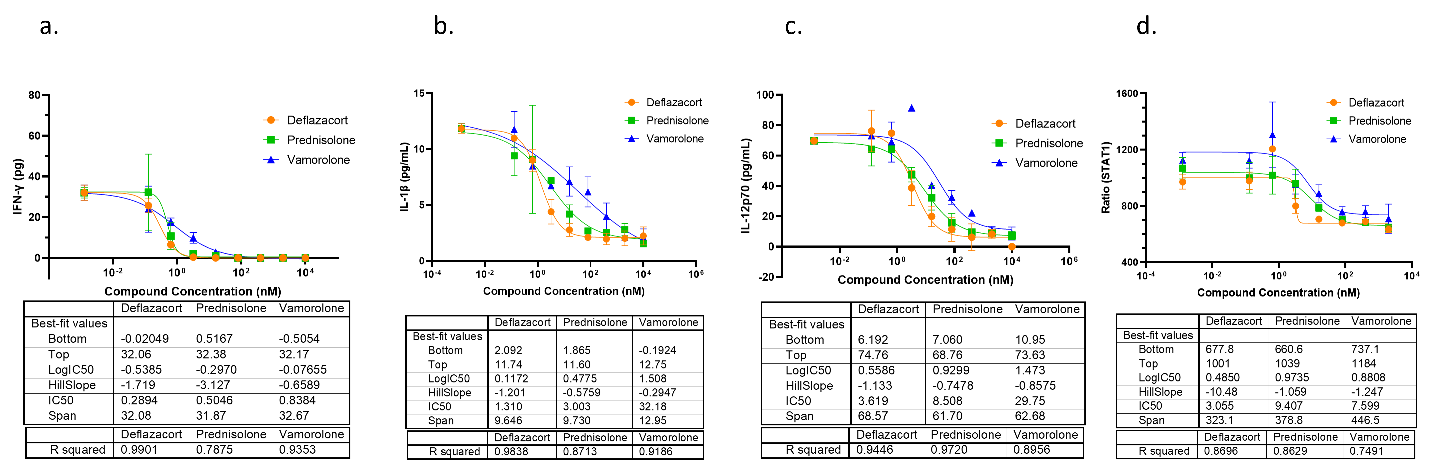


**Supplementary Table S3: Measured PK Data in Mice (Experimental) and in Patients (Published)**

| **Drug** | **Dose (mg/kg)** | **AUC (Day 1) hr.ng/mL** | **Cmax ng/mL** |
| --- | --- | --- | --- |
| **HUMAN (published)** | | | |
| Vamorolone^a^ | 6 | 3279 | 856 |
| Vamorolone^a^ | 2 | 761 | 199 |
| Prednisolone^b^ | 0.35 | 3301 | 659 |
| Prednisolone^c^ | 0.75 | (~7073) | (~705) |
| Emflaza^d^ | 0.9 | 567, 630  (~599, average) | 329 |
|  |  |  |  |
| **MOUSE (experimental, this study)** | | | |
| Vamorolone (DBA) | 45 | 34090 | 3470 |
| Vamorolone (DBA) | 30 | 26590 | 2700 |
| Vamorolone (DBA) | 15 | 9350 | 1980 |
| Prednisolone (DBA) | 5 | 3929 | 485 |
| Deflazacort^b^ (DBA) | 4 | 357 | 214 |

^a^ Conklin LS, Damsker JM, Hoffman EP, Jusko WJ, Mavroudis PD, Schwartz BD, Mengle-Gaw LJ, Smith EC, Mah JK, Guglieri M, Nevo Y, Kuntz N, McDonald CM, Tulinius M, Ryan MM, Webster R, Castro D, Finkel RS, Smith AL, Morgenroth LP, Arrieta A, Shimony M, Jaros M, Shale P, McCall JM, Hathout Y, Nagaraju K, van den Anker J, Ward LM, Ahmet A, Cornish MR, Clemens PR. Phase IIa trial in Duchenne muscular dystrophy shows vamorolone is a first-in-class dissociative steroidal anti-inflammatory drug. Pharmacol Res. 2018 Oct;136:140-150. doi: 10.1016/j.phrs.2018.09.007. Epub 2018 Sep 13. PMID: 30219580; PMCID: PMC6218284.

^b^ 20 mg/57 kg = 0.35 mg/kg in adults; AUC_0-12_ is reported and represented more than 80% of the AUC_INF_; averaged the AUC and C_max_ from two different formulations; Bashar T, Apu MNH, Mostaid MS, Islam MS, Hasnat A. Pharmacokinetics and Bioavailability Study of a Prednisolone Tablet as a Single Oral Dose in Bangladeshi Healthy Volunteers. Dose Response. 2018 Jul 25;16(3):1559325818783932. doi: 10.1177/1559325818783932. PMID: 30083083; PMCID: PMC6073839.

^c^ Assumed linear increase in AUC and C_max_ from 0.25 to 0.75 mg/kg. This may be an overestimate. However, there is support for linearity in Al-Habet S, Rogers HJ. Pharmacokinetics of intravenous and oral prednisolone. Br J Clin Pharmacol. 1980 Nov;10(5):503-8. doi: 10.1111/j.1365-2125.1980.tb01796.x. PMID: 7437263; PMCID: PMC1430141.

^d^ <https://www.accessdata.fda.gov/drugsatfda_docs/nda/2017/208684,208685Orig1s000ClinPharmR.pdf>

**Supplementary Table S4: Data from mdx mice treated for 2-weeks (Study 1) and comparison to predicted human exposure**

| **Group** | **Drug** | **Dose PO mg/kg** | **% decrease in fraction of lymphocytes at Day 14 from mdx vehicle** | **% increase in fraction of PMNs at Day 14** | **Ratio to human exposure based on Cmax**  ***ESTIMATED*** | **Ratio to human exposure based on AUC**  ***ESTIMATED*** |
| --- | --- | --- | --- | --- | --- | --- |
| 1 | Vehicle/ wt |  |  |  |  |  |
| 2 | Vehicle |  |  |  |  |  |
| 3 | deflazacort | 1.25 | (1%↑) | 3% |  |  |
| 4 | deflazacort | 2.5 | 5% | 32%* |  |  |
| 5a | deflazacort | 4 |  |  | 0.7^a^ | 0.6 ^a^ |
| 5b | deflazacort | 5 | 9%* | 41%* | 0.8 ^b^ | 0.8 ^b^ |
| 6 | deflazacort | 15 | 19%**** | 82%**** |  |  |
| 7 | vamorolone | 15 | (0.5%↑) | 7% | 2^C^ | 3^C^ |
| 7 | vamorolone | 30 | 5% | 27% | 3^C^ | 8^C^ |
| 9 | vamorolone | 45 | 14%** | 70%** | 4^C^ | 10^C^ |
| 10 | pred | 1.2 | 17%** | 64%** |  |  |
| 11 | pred | 2.4 | 18%** | 81%*** |  |  |
| 12 | pred | 5.0 | 21%*** | 92%**** | (≥0.6^d^) | (≥0.7^d^) |

Calculations for determining the ratio of the mouse exposure to the human clinical exposure was performed utilizing data reported in Supplementary Table S3 and is described in more detail below.

^a^ 4 mg/kg: C_max_ = 214 in mice/ 329 ng/mL in human = 0.65; AUC = 357 in mice/ 599 hr.ng/mL in human = 0.6

^b^5 mg/kg: Based on a PK in mice at 4 mg/kg, assumed dose-linearity to 5 mg/kg: C_max_ at 4 mg/kg =214 ng/mL, AUC = 357 hr.ng/mL; predict at 5 mg/kg C_max_ is 214 x 5/4 = 267.5, AUC is 357x5/4 = 446 hr.ng/mL

C_max_ = 267.5 in mice/ 329 ng/mL in human = 0.81

^c^Based on a clinical dose of 6 mg/kg vamorolone:

15 mg/kg: Cmax =1980 in mice/856 ng/mL in humans = 2.3; AUC of 9350 in mice/ 3279 hr.ng/mL in human = 2.85

30 mg/kg: Cmax = 2700 in mice/856 ng/mL in humans = 3.1; AUC of 26590 in mice/ 3279 hr.ng/mL in human = 8.11

45 mg/kg: Cmax = 3470 in mice/856 ng/mL in humans = 4.1; AUC of 34090 in mice/ 3279 hr.ng/mL in human = 10.3

^d^ A reference for exposure of prednisolone in DMD at a dose of 0.75 mg/kg was not identified. Therefore, using reported data for 0.35 mg/kg (Bashar 2018), it was assumed that exposure increased linearly. This is likely an overestimation of the human exposure and therefore an underestimate of the ratio of the mouse to the human dose.

Based on this estimated exposure in humans at 0.75 mg/kg:

5 mg/kg: Cmax = 485 in mice/ ~705 ng/mL in humans = (≤0.69); AUC= 3929 in mice/ 7073 hr.ng/mL =(≤0.55)
